# Effects of pharmacist-led home visit services and factors influencing medication adherence improvement

**DOI:** 10.1371/journal.pone.0314204

**Published:** 2024-11-22

**Authors:** Hwayoung Ahn, Bo-Kyung Byun, Tae-Hoon Lee, Dong-Won Kang, Sun-Kyeong Park

**Affiliations:** 1 College of Pharmacy, The Catholic University of Korea, Bucheon, Gyeonggi-do, South Korea; 2 Division of Outcomes Research and Quality, Department of Surgery, Penn State College of Medicine, Hershey, Pennsylvania, United States of America; Complete HEOR Solutions, UNITED STATES OF AMERICA

## Abstract

**Background:**

The use of medicines is crucial in treatment, but nonadherence poses an important challenge, particularly when managing polypharmacy and long-term conditions. Pharmacist-led home visit services offer a promising solution to enhance the outcomes associated with medication use. However, the effects and the factors contributing to this improvement remain unclear.

**Objective:**

This study assessed the effects of pharmacist-led home visit services on medication adherence and general medication knowledge. Additionally, we analyzed the factors associated with improved medication adherence.

**Methods:**

Face-to-face, pharmacist-led home visit services were conducted via opportunistic sampling in community settings. Data were collected between 2017 and 2019. The program included participants aged ≥65 years or taking ten or more medications, in need of care, and who were willing to participate. We estimated the effects of the program by improved medication adherence and general knowledge about taking medications. Medication adherence was measured using the Morisky Scale. We used McNemar’s test to evaluate the statistical differences in outcomes before and after consultation. To identify factors influencing improvements in medication adherence, odds ratios (OR) with 95% confidence intervals (CI) were calculated using multivariate logistic regression with adjustments for covariates.

**Results:**

Among the 1,194 participants in the program, 874 were included in the analysis. Pharmacist-led home visit services improved both medication adherence (from 69.2% to 85.8%) and mean scores for general knowledge of taking medications (from 65.3% to 89.5%). Participants aged ≥70 years showed less improvement in overall adherence than those aged <70 years (OR = 0.51; 95% CI = 0.317–0.817). The program was significantly more effective at improving adherence for participants with higher level of medication knowledge (OR = 2.93; 95% CI = 1.78–4.81) compared to those with lower level of knowledge.

**Conclusion:**

These quantitative findings highlight the importance of pharmacist-led interventions and suggest a framework for future programs about medication management.

## Introduction

The prescription of pharmaceutical medicines is among the most common interventions because medicines play an essential role in treatment [1]. Despite its importance, non-adherence can be a significant barrier to achieving optimum outcomes with appropriately prescribed medicines [1]. Medication adherence may worsen when treating particularly long-term conditions or when multiple medications are used [1–4]. Considering that the older adults take several medications over a long time owing to complex chronic diseases, the need for medication management and review by experts is being raised [5]. In addition, low-income patients taking polypharmacy are at higher risk of health deterioration and disability due to the low accessibility to health care. Therefore, expert drug management strategies are required [5].

Pharmacists are experts in drug management because pharmacist-led medication reviews have been reported to reduce polypharmacy and medication-related problems [5–7]. Further, it has been proposed that conducting medication reviews at home could help recognize problems related to medicines and create a more engaging environment for creating awareness regarding medication [8]. Additionally, pharmacist-led home visits enable a comprehensive review of medications prescribed by different healthcare providers, significantly mitigating the risks associated with polypharmacy such as medication duplication and interactions. Home pharmaceutical services are provided in various ways in different countries. Australia has provided government-funded home management assessment programs. Through the program, pharmacists comprehensively review medications in patients’ homes and collaborate with general practitioners to improve patient health outcomes and prevent drug-related problems [8]. Home pharmaceutical care has been provided in Japan since 1994. In this program, pharmacists visit patients’ homes to administer medications and check medication reviews [9]. Pharmacist-led home visit services have demonstrated significant improvements in medication adherence, uptake rates, and quality of life [10]. In South Korea, pharmacist-led home visit services have been provided since 2014, in cooperation with pharmacy associations and local governments. For example, the Gyeonggi-do Pharmacy Association implemented a home-based pharmaceutical program for older adult patients with chronic diseases or polypharmacy since 2017 [11, 12].

Home-based pharmaceutical services improve medication adherence in older adult patients [8]. A community pharmaceutical care program was found to change medication adherence from non-adherence to adherence in a significantly higher proportion of intervention patients than in control patients (13.4% vs. 9.1%) [13]. Although the positive effects of these services have been demonstrated, it remains unclear what factors contribute to improved adherence. Factors influencing adherence, such as patient-, disease-, and medication-specific characteristics, have been identified in previous studies [14, 15]. However, the factors influencing adherence to home pharmaceutical services have not yet been identified. Understanding the factors that improve adherence to home-based pharmaceutical services is critical for targeting specific populations and planning programs. Therefore, this study aimed to evaluate the effects of pharmacist-led home visit services and examine the factors affecting improving medication adherence among program participants in South Korea, with a focus on older adults.

## Methods

### Program overview

Participants in the home-based pharmaceutical program were recruited annually from 2017 to 2019 through public health centers and the Gyeonggi-do Pharmacy Association. Participants who met the following criteria were included: 1) age ≥65 years or older, or those taking ten or more drugs for chronic or overlapping conditions; 2) living alone or in need of care through senior support centers, public health centers, or pharmacies; and 3) willing to participate in the program. Participants with missing information on gender, age, or first and last responses of medication adherence or general knowledge about taking medications were excluded.

Pharmacists with prior experience as Medication Safety program instructors were given preference when recruiting from the Gyeonggi Province Pharmacists’ Association. Visiting pharmacists were trained to standardize education and counseling for the participants prior to the program. The training was conducted in two phases: the first session took place before the program began, and the second was a follow-up session during the program’s execution. The first phase of pre-training (3 hours) covered the meaning and objectives of this program, the roles and services of home-visiting pharmacists, international case studies, and practical training in home medicine review. The training also included instructions for documenting consultation forms. The second phase of follow-up training (three hours) focused on an overview of geriatric pharmacy services, specific care for elderly patients, sharing progress by local divisions, and discussing exemplary cases.

The program consisted of at least two in-person counseling sessions. In 2017, pharmacists conducted in-person counseling at the participants’ homes for the first and fifth sessions and telephone counseling for middle sessions at 20-day intervals. In 2018 and 2019, pharmacists conducted in-person counseling at the participants’ homes three times at 30-day intervals. Each counseling session examined changes in medication adherence compared to the previous visit and continued to educate participants on the importance of managing health care and medication adherence. The counseling results were recorded for each session.

The participants signed a consent form to participate in the home medicine review program and agreed that their personal information could be anonymized and used for research purposes. The Gyeonggi-do Pharmacy Association anonymized and de-identified all personal information, sharing it with the researchers. When the researchers accessed anonymized secondary data for analysis in February 2024, the Institutional Review Board of the Catholic University of Korea waived the requirement to obtain additional informed consent for research participation (IRB No. 1040395-202304-901).

### Health belief model

This study was anchored in the Health Belief Model (HBM), which is widely used model for understanding health-related behaviors [13, 16, 17] and medication adherence [18, 19]. When employing the HBM, we defined health-related behavior as an improvement in medication adherence. The current model included sex and age as defined demographic variables and medication assistance as a socio-psychological variable. We conceptualized communication skills, health literacy, and memory as elements of perceived susceptibility and severity, recognizing their influence on individuals’ awareness and evaluation of health risks. General knowledge of medication was treated as a perceived benefit, highlighting its role in fostering positive health behaviors. The number of diseases was considered a perceived barrier, reflecting the potential consequences of non-adherence. Self-efficacy was defined as confidence in health management. In this study, we could not incorporate cues to action because of a lack of relevant data.

### Measurement for covariates

The baseline characteristics of the participants were recorded during the initial counseling session. Communication skills, health literacy, memory, and confidence in one’s health were measured on a five-point Likert scale. The five-point Likert scales were divided into two groups to facilitate statistical analysis, with "very good" and "good" in one group and "neutral,” "bad,” and "very bad" in the other. General knowledge about taking medications was evaluated using the following seven questions: 1) Should not you take someone else’s prescription drugs even if you have similar symptoms?; 2) Should not you change the way you take your medications without consulting your doctor or pharmacist?; 3) Should you discard expired medicines immediately?; 4) Are you aware that some medications come in different forms even if they have the same ingredients?; 5) Awareness regarding consuming milk with medication as it may interact with it; 6) Awareness regarding difference in side effects of drugs in different individuals; 7) Knowledge regarding consuming alcohol and tobacco as they may interfere with the efficacy of the medication. Responses were binary, with "yes" scoring 1 and "no" scoring 0; higher scores indicated better general knowledge.

### Measurement for effects

The program’s effectiveness was assessed by analyzing the increase in the proportion of participants who demonstrated improved medication adherence and enhanced general medication knowledge from their initial visit to their last visit. This analysis was performed by determining the proportion of participants who provided correct responses to questions on medication adherence (hereafter defined as adherence rate), both in specific areas and overall, as well as to questions on general medication knowledge at both initial and final visits. Medication adherence was assessed using the Morisky Medication Adherence Scale, which consists of four items divided into two domains [20]. The four items cover four ways that drug errors of omission can occur, with “forgetting” or “carelessness” being categorized into motivation domain and “stopping the drug when feeling better” or “starting the drug when feeling worse” being categorized into knowledge domain [20]. First, the motivation domain included 1) "Do you ever forget to take your medicine?", and 2) "Are you careless at times about taking your medicine?" The knowledge domain included: 3) "When you feel better, do you sometimes stop taking your medicine?" and 4) "Sometimes, if you feel worse when you take the medicine, do you stop taking it?" Responses were binary, with "yes" scoring 0 and "no" scoring 1; higher scores indicated better adherence. Improvement in adherence was identified when a participant’s last visit response indicated a positive change in at least one question within each domain. Overall, improvement in adherence was acknowledged if either the motivation or knowledge domains showed betterment. Regarding medication knowledge, each correct response was awarded one point, with higher total scores reflecting more substantial knowledge.

### Statistical analysis

We included only participants that had at least two in-person counseling sessions, including the initial visit. Among them, we excluded populations with missing values for sex and age, which were the main baseline characteristics, as well as missing data on medication adherence and general knowledge about taking medications. Categorical variables are presented as numbers and percentages, whereas continuous variables are presented as means and standard deviations (SD). We used the McNemar test to compare differences in medication adherence and general medication knowledge before and after consultation. Univariate and multivariate logistic regression analyses were performed for each covariate to identify factors influencing adherence improvement. Odds ratios (OR) and 95% confidence intervals (CI) were estimated using logistic regression models. We adjusted for covariates, including age group, sex, medication assistance, communication skills, health literacy, memory, confidence in health, general knowledge about taking medications, and number of diseases in the multivariate logistic regression. Statistical significance was set at p<0.05. Statistical analyses were performed using SAS version 9.4 (SAS Institute Inc., Cary, North, USA).

## Results

### Baseline characteristics

Of the 1,194 participants, 320 were excluded due to missing information on sex, age, or first and last responses of medication adherence or general knowledge about taking medications, leaving a total of 874 participants included in the analysis (S1 Fig). Among them, 71.7% were female, and 83.1% were aged ≥70 years. The proportion of participants living alone (70.1%) was higher than that of participants living with their families and/or caregivers (24.4%). Approximately 71% of the participants had good communication skills, about half had poor health literacy and memory, and about 82% had poor self-efficacy. The mean total score for general knowledge about medications was 1.74 (SD = 1.64). More than half of the participants (54.9%) had five or more diseases (Table 1).

**Table 1:** Baseline characteristics of home-based pharmaceutical services participants.

Baseline characteristic, n (%)	Participants (n = 874)
Sex	
Male	247 (28.3)
Female	627 (71.7)
Age group	
<70 years	148 (16.9)
≥70 years	726 (83.1)
Medication assistant	
Family, Caregiver	213 (24.4)
Living alone	613 (70.1)
Missing	48 (5.5)
Communication skill [Mean (SD)]	2.61 (0.66)
Poor, Neutral	252 (28.8)
Good	619 (70.8)
Missing	3 (0.4)
Health literacy [Mean (SD)]	2.33 (0.78)
Poor, Neutral	411 (47.0)
Good	453 (51.8)
Missing	10 (1.1)
Memory [Mean (SD)]	2.36 (0.71)
Poor, Neutral	435 (49.8)
Good	435 (49.8)
Missing	4 (0.4)
Total score of general knowledge about taking medications [Mean (SD)]	1.74 (1.64)
0 Point	256 (29.3)
≥1 Point	618 (70.7)
Number of diseases [Mean (SD)]	5.20 (2.57)
<5	394 (45.1)
≥5	480 (54.9)
Self-efficacy for health [Mean (SD)]	1.71 (0.74)
Poor, Neutral	716 (81.9)
Good	145 (16.6)
Missing	13 (1.5)

Abbreviations: SD, standard deviation.

### Effects of pharmacist home visit program

**Fig 1:**
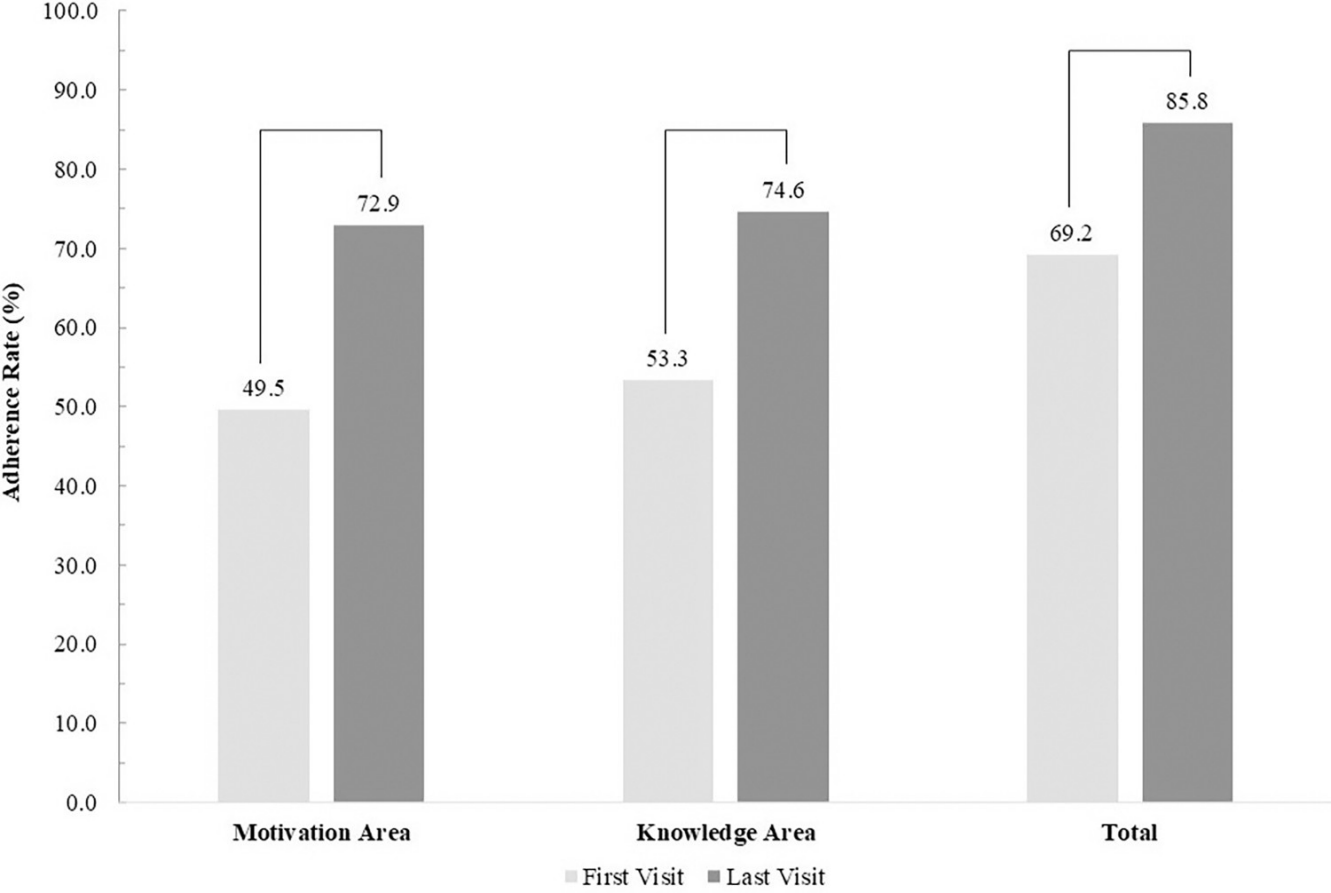
Comparison of medication adherence rate between the first visit and last visit; P<0.001*.

**Fig 2:**
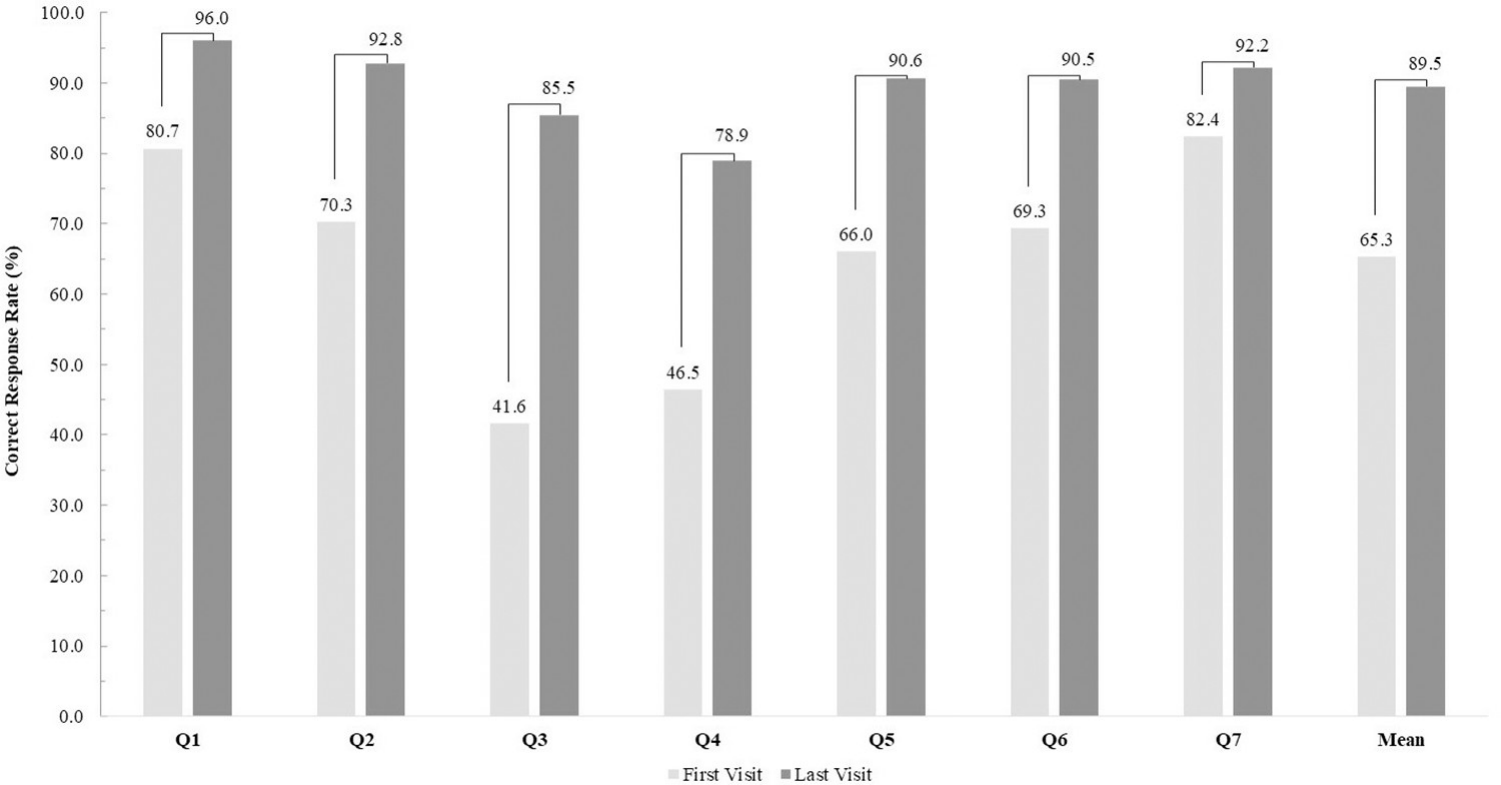
Comparison of patient’s general drug knowledge between the first visit and last visit; P<0.001*. The seven questions about general drug knowledge are as follows: Q1. Should not you take someone else’s prescription drugs even if you have similar symptoms?/ Q2. Should not you change the way you take your medications without consulting your doctor or pharmacist?/ Q3. Should you discard expired medicines immediately?/ Q4. Are you aware that some medications come in different forms even if they have the same ingredients?/ Q5. Awareness regarding consuming milk with medication as it may interact with it./ Q6. Awareness regarding difference in side effects of drugs in different individuals./ Q7. Knowledge regarding consuming alcohol and tobacco as they may interfere with the efficacy of the medication. Patients who answered "Yes" to each question were considered to have high general drug knowledge.

In the areas of motivation, knowledge, and total adherence, adherence rates increased at the final visit compared to the first visit, regardless of the area (Fig 1). In the motivational area, the adherence rate significantly increased from 49.5% at the first visit to 72.9% at the final visit (p<0.001). Regarding knowledge, the adherence rate significantly increased from 53.3% at the first visit to 74.6% at the final visit (p<0.001). The adherence rate in the total adherence area increased significantly from 69.2% at the first visit to 85.8% at the final visit (p<0.001). Regarding general knowledge about taking medication, the correct response rates significantly increased at the final visit compared with the first visit for all questions (Fig 2). Specifically, for the third question about discarding the expired medicines, the proportion of respondents answering correctly increased approximately two-fold, from 41.6% to 85.5% (p<0.001). The second-highest improvement in general knowledge was observed in question four ("Are you aware that some medications come in different forms even if they have the same ingredients?"). The rate of correct responses increased from 46.5% during the first visit to 78.9% during the final visit.

### Influencing factors on improving medication adherence

The results of univariate and multivariate logistic regression analyses are shown in Tables 2 and 3, respectively. Participants aged ≥70 years showed less improvement in overall adherence than those aged <70 years in both univariate (OR = 0.58, 95% CI = 0.38–0.87) and multivariate logistic regression (OR = 0.51, 95% CI = 0.32–0.82). In the univariate logistic regression, participants with poor or neutral health literacy showed better adherence improvement that those with good health literacy in the motivation (OR = 1.51, 95% CI = 1.11–2.05) and overall areas (OR = 1.46, 95% CI = 1.04–2.05). Health self-efficacy was relevant only in motivation, with an OR of 1.61 (95% CI = 1.03–2.53) for poor or neutral self-efficacy compared to good self-efficacy. Participants with five or more diseases showed better total adherence improvement compared to those with fewer than five diseases (OR = 1.47, 95% CI = 1.04–2.08). However, they were not statistically significant in multivariate logistic regression analysis. Memory and general knowledge of medication were significantly associated with improvement in medication adherence, even after adjusting for covariates in the multivariate logistic regression. Having poor or neutral memory was associated with better adherence improvement compared to having good memory in the motivation (OR = 1.49, 95% CI = 1.00–2.21) and overall areas (OR = 1.71, 95% CI = 1.09–2.69). Compared to participants with a knowledge score of 0, those with a score of ≥1 had OR of 1.98 (95% CI = 1.33–2.94), 3.25 (95% CI = 2.07–5.09), and 2.93 (95% CI = 1.78–4.81) for the motivation, knowledge, and overall areas, respectively.

**Table 2:** The results of univariate logistic regression of improvement in medication adherence.

Covariate	Univariate analysis					
	Motivation Area		Knowledge Area		Overall	
	OR (95% CI)	*P value*	OR (95% CI)	*P value*	OR (95% CI)	*P value*
Gender						
Female	1.04 (0.74–1.46)	0.8231	1.05 (0.74–1.48)	0.7972	0.88 (0.61–1.27)	0.5021
Male	[reference]		[reference]		[reference]	
Age group						
≥70 years	0.72 (0.49–1.06)	0.0965	0.89 (0.59–1.33)	0.5625	0.58 (0.38–0.87)	0.0088
<70 years	[reference]		[reference]		[reference]	
Medication assistant						
Family, Caregiver	1.00 (0.70–1.42)	0.9734	0.85 (0.58–1.22)	0.3709	0.82 (0.54–1.23)	0.3380
Live alone	[reference]		[reference]		[reference]	
Communication skill						
Poor, Neutral	1.24 (0.89–1.72)	0.2007	1.32 (0.95–1.84)	0.1037	1.31 (0.91–1.88)	0.1454
Good	[reference]		[reference]		[reference]	
Health literacy						
Poor, Neutral	1.51 (1.11–2.05)	0.0086	1.15 (0.84–1.57)	0.3723	1.46 (1.04–2.05)	0.0307
Good	[reference]		[reference]		[reference]	
Memory						
Poor, Neutral	1.65 (1.21–2.24)	0.0015	1.32 (0.97–1.80)	0.0828	1.83 (1.30–2.59)	0.0006
Good	[reference]		[reference]		[reference]	
Total score of general knowledge about taking medications						
≥1 Points	2.05 (1.42–2.97)	0.0001	3.05 (2.02–4.62)	<0.0001	2.78 (1.77–4.38)	<0.0001
0 Point	[reference]		[reference]		[reference]	
Number of diseases						
≥5	1.45 (1.06–1.97)	0.0200	1.60 (1.17–2.20)	0.0036	1.47 (1.04–2.08)	0.0286
<5	[reference]		[reference]		[reference]	
Self-efficacy for health						
Poor, Neutral	1.61 (1.03–2.53)	0.0372	1.23 (0.80–1.89)	0.3552	1.41 (0.86–2.30)	0.1710
Good	[reference]		[reference]		[reference]	

Abbreviations: OR, odds ratio, CI, Confidence interval.

**Table 3:** The results of multivariate logistic regression of improvement in medication adherence.

Variables	Multivariate analysis					
	Motivation Area		Knowledge Area		Overall	
	OR (95% CI)	*P value*	OR (95% CI)	*P value*	OR (95% CI)	*P value*
Gender						
Female	1.06 (0.72–1.56)	0.7709	1.08 (0.73–1.59)	0.7170	1.00 (0.65–1.54)	0.9887
Male	[reference]		[reference]		[reference]	
Age group						
≥70 years	0.69 (0.45–1.07)	0.0977	0.80 (0.51–1.26)	0.3295	0.51 (0.32–0.82)	0.0052
<70 years	[reference]		[reference]		[reference]	
Medication assistant						
Family, Caregiver	0.99 (0.68–1.43)	0.9450	0.85 (0.58–1.25)	0.4089	0.82 (0.54–1.26)	0.3606
Live alone	[reference]		[reference]		[reference]	
Communication skill						
Poor, Neutral	0.83 (0.54–1.26)	0.3842	1.37 (0.88–2.13)	0.1615	1.01 (0.63–1.61)	0.9801
Good	[reference]		[reference]		[reference]	
Health literacy						
Poor, Neutral	1.36 (0.91–2.03)	0.1398	0.85 (0.55–1.29)	0.4381	1.14 (0.72–1.80)	0.5704
Good	[reference]		[reference]		[reference]	
Memory						
Poor, Neutral	1.49 (1.00–2.21)	0.0496	1.19 (0.79–1.79)	0.4111	1.71 (1.09–2.69)	0.0193
Good	[reference]		[reference]		[reference]	
Total score of general knowledge about taking medications						
≥1 Points	1.98 (1.33–2.94)	0.0007	3.25 (2.07–5.09)	<0.0001	2.93 (1.78–4.81)	<0.0001
0 Point	[reference]		[reference]		[reference]	
Number of Diseases						
≥5	1.41 (1.02–1.97)	0.0395	1.64 (1.17–2.31)	0.0044	1.38 (0.96–1.99)	0.0924
<5	[reference]		[reference]		[reference]	
Self-efficacy for health						
Poor, Neutral	1.26 (0.76–2.10)	0.3735	1.04 (0.63–1.71)	0.8786	0.96 (0.55–1.69)	0.8963
Good	[reference]		[reference]		[reference]	

Abbreviations: OR = odds ratio, CI = Confidence interval.

## Discussion

This study evaluated the effects of pharmacist-led home visit services and demonstrated that these services improved medication adherence and knowledge. Additionally, this study analyzed the factors associated with improvement in medication adherence using logistic regression. Our study found that patients’ general knowledge about taking medication significantly increased after pharmacist-led home visits. While the mean baseline general medication knowledge score was low, at 1.74 (SD = 1.64), the pharmacist-led home visit service improved all seven general knowledge items. Knowledge was also identified as a key factor significantly affecting improvement in medication adherence. Better medication knowledge indicates that patients have a better understanding of their medications, regimens, benefits, and risks, which can also correlate with higher medication adherence [21]. Therefore, it is necessary to consider improving education regarding general knowledge of medication use in pharmacist services. In addition, participants with poor or neutral memory were likely to have improved medication adherence compared to those with good memory. This suggests that pharmacist-led medication therapy is more effective in improving medication adherence in older adults with memory impairment than in those with good memory. Patients with poor memory are more likely to forget when and how to take their medications, making it difficult to achieve high adherence rates. For these patients, pharmacist-led home visit services can help them remember how to take their medications and ensure they are taking them correctly, leading to better adherence. In the univariate logistic regression analysis, health literacy was also associated with improvement in adherence. Since participants with poor or neutral health literacy may have low medication knowledge, pharmacist-led home visiting services may be positively associated with improved medication adherence for these participants; however, the association disappeared after adjustment for covariates. Health self-efficacy and number of diseases were also associated with improvement in adherence but were not statistically significant after adjusting for covariates.

While direct comparisons are constrained due to lack of medication adherence research on pharmacist-led home visit services, our findings are comparable to several previous studies in other settings. Previous studies reported several categories affecting medication adherence [22, 23], including patient factors such as age, education, and self-efficacy [19]. Notably, older patients were more likely to have poorer medication knowledge, and medication knowledge was positively associated with adherence in patients aged ≥80 years [24]. This is consistent with our findings that general knowledge about taking medication has a positive impact on improving medication adherence.

On the other hand, previous studies showed that self-efficacy improves medication adherence [25, 26], but our study did not find a significant effect of self-efficacy. This difference may be due to variations in target populations, interventions, and study designs. For example, unlike our study, the previous studies were randomized controlled trials and included populations aged ≥30 years and with a single disease. Further, the pharmacist-led interventions in those studies enhanced socio-psychological support, whereas the interventions in our study were more focused on education about medication adherence and knowledge. To increase the effectiveness of self-efficacy in improving medication adherence, social and psychological support should also be strengthened in home-based pharmacy services.

Age and having a medication assistant were not associated with improved adherence in our study. However, the majority of the demand for these services came from adults older than 70 years or those living alone. Older age and living alone have been reported to be negatively associated with medication adherence [14]. Furthermore, older adults living alone are more susceptible to illnesses [27, 28] and are less likely to receive preventive care [29]. Although our study found no association between age or the presence of a medication assistant and adherence improvement, services should be planned considering the adherence behavior and health status of service users.

With the global increase in the older adult population, concerns about polypharmacy are intensifying [5]. In addition, poor medication adherence in this population can result from complex regimens, communication gaps between healthcare providers, and improper medication storage [14]. Therefore, various countries have initiated homecare services to address medication-related challenges. For instance, Australia’s Department of Health runs programs, such as Home Medicines Review and Residential Medication Management Review, enabling pharmacists to review patient medications at home or in care settings and collaborate with doctors for effective medication plans [8]. Japan has adopted a comprehensive community care system in which pharmacists offer home care guidance based on pharmaceutical care plans [9]. Similarly, Taiwan’s National Health Insurance (NHI) Administration employs the "NHI Pharma cloud system" to facilitate home visits by family doctors and pharmacists, ensuring real-time medication tracking and preventing prescription overlaps [30].

In South Korea, the proportion of older adult population has been increasing, reaching 17.47% in 2022, and it is expected to become an ultra-aged society by 2025, exceeding 46.4% by 2070 [20]. Recognizing this problem, the Pharmaceutical Association of Gyeonggi-do has provided pharmacist home-visit services, evaluated in this study. In addition, in 2018, South Korea initiated a pilot project to support proper medication use through the National Health Insurance Service [31]. This project demonstrated improvements in medication adherence and general knowledge through pharmacist-led patient education. The improvements in both programs were interpreted as a positive change in medication awareness and understanding of their diseases due to the counseling and education provided by visiting pharmacists. However, unlike our study, which targeted low-income recipients of medical aid with chronic diseases on polypharmacy, this project selected individuals aged 30–100 years with four chronic diseases (hypertension, diabetes, cardiovascular disease, and chronic kidney disease) residing in nine pilot areas. Reports suggest that patients on polypharmacy in low-income groups require medication management from professionals because of their low accessibility to healthcare, which leads to higher risks of health deterioration and disability [32]. A study targeting individuals aged ≥65 years in South Korea showed that medical aid recipients had a higher incidence of chronic diseases and poorer subjective perceptions of health than health insurance subscribers [33]. Therefore, the tailored medication counseling by pharmacists to ensure safe medication would be important considered in various policies.

Our study is the first to analyze the factors influencing improvement in medication adherence based on pharmacists’ home-visit data. Pharmacist-led home visit services were provided in cooperation with pharmacy associations and local governments, with standardized intervention protocols and the systematic training of pharmacists. We expect that this study will contribute to the advancement of pharmaceutical care practices. We showed the results by offering tailored counseling and education during visits to the residences of older adults managing chronic illnesses and polypharmacy in collaboration with local communities and municipalities. Identifying the determinants of adherence to home-based pharmaceutical care is of paramount importance. Given that resources and time are limited, identifying those most susceptible to non-adherence will allow for more targeted education and support. Furthermore, our findings can be instrumental in determining the eligibility for home pharmaceutical care, ensuring that those who stand to benefit the most are prioritized. In a context in which the need for home pharmaceutical services is growing, the results of our study are expected to provide a foundation for deciding service beneficiaries and planning programs. This approach enhances the overall efficacy of home pharmaceutical interventions and ultimately contributes to improved health outcomes in vulnerable populations.

Several limitations must be acknowledged when interpreting our results. First, our analysis had limited statistical power due to the small sample size, which potentially affected the robustness and generalizability of our findings. Further studies with larger populations are necessary to validate our results and draw more definitive conclusions. Second, there were limitations due to the use of secondary data sources. Our study did not consider all factors related to medication adherence or the health belief model. Variables that significantly influenced medication adherence were also excluded. Medication adherence is a complex behavior influenced by various factors; therefore, future research should include a broader range of variables. Additionally, we integrated annual data instead of analyzing continuous data, which could have led to a potential bias. Fourth, we were unable to determine the sustainability of improvements in adherence knowledge owing to the limited study design. However, given that high adherence is positively associated with persistence [34, 35], improving adherence through pharmacist-led home visit services is expected to improve persistence. Fifth, we collapsed the Likert scale data into two groups, which was necessary because of differences in scale use across years and uneven response distributions. Although this approach helped stabilize the analysis, it may have compromised the sensitivity of the measurements and the nuances of the data. Finally, the generalizability of our findings to other groups may be limited due to the specific demographic and clinical characteristics of our study population. Considering these limitations, the extrapolation of our findings to other populations or settings should be performed with caution.

## Conclusion

Pharmacist-led home visit services improved both the participants’ medication adherence and knowledge of medication. The population aged <70 years with poor memory and a higher level of medication knowledge at baseline showed better improvement in medication adherence even after adjusting for covariates. Health literacy, health self-efficacy, and number of diseases were associated with improvement in adherence, but these associations disappeared after adjusting for covariates. Our findings provide a basis for determining the eligibility for home pharmaceutical care, ensuring that those who stand to benefit the most are prioritized. Additionally, we suggest providing education of medication knowledge, a key factor in increasing medication adherence, in pharmacist services to improve adherence rates further.

## Supporting information

S1 Fig The process of selecting study participants. (JPG)
