# Supplementary figures and images for: Effects of pharmacist-led home visit services and factors influencing medication adherence improvement
Source: PLoS One. 2024 Nov 22;19(11):e0314204. doi: 10.1371/journal.pone.0314204 (PMC11584129; doi:10.1371/journal.pone.0314204)

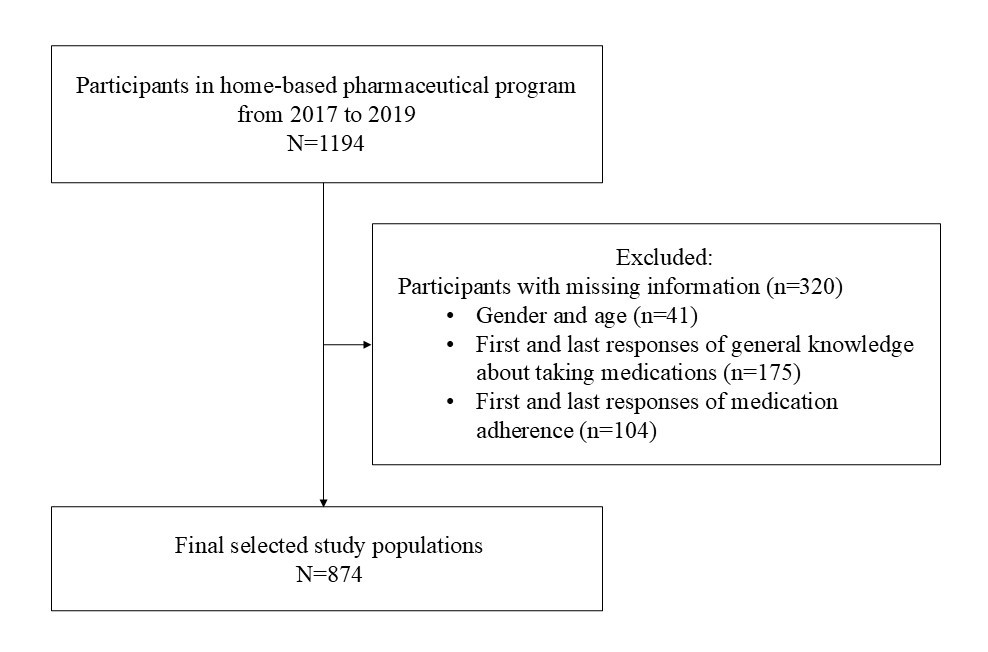

Supplement: S1 Fig — (JPG) [file pone.0314204.s001.jpg]
